# Chronology of Fabry-Perot Interferometer Fiber-Optic Sensors and Their Applications: A Review

**DOI:** 10.3390/s140407451

**Published:** 2014-04-24

**Authors:** Md. Rajibul Islam, Muhammad Mahmood Ali, Man-Hong Lai, Kok-Sing Lim, Harith Ahmad

**Affiliations:** Photonics Research Centre, University of Malaya, Kuala Lumpur 50603, Malaysia; E-Mails: md.rajibul.islam@gmail.com (M.R.I.); engrmahmoodnaz@gmail.com (M.M.A.); a131859.ukm@gmail.com (M.-H.L.); harith@um.edu.my (H.A.)

**Keywords:** interferometer, Fabry-Perot Interferometer (FPI), FPI fabrication techniques, FPI sensing applications

## Abstract

Optical fibers have been involved in the area of sensing applications for more than four decades. Moreover, interferometric optical fiber sensors have attracted broad interest for their prospective applications in sensing temperature, refractive index, strain measurement, pressure, acoustic wave, vibration, magnetic field, and voltage. During this time, numerous types of interferometers have been developed such as Fabry-Perot, Michelson, Mach-Zehnder, Sagnac Fiber, and Common-path interferometers. Fabry-Perot interferometer (FPI) fiber-optic sensors have been extensively investigated for their exceedingly effective, simple fabrication as well as low cost aspects. In this study, a wide variety of FPI sensors are reviewed in terms of fabrication methods, principle of operation and their sensing applications. The chronology of the development of FPI sensors and their implementation in various applications are discussed.

## 1. Introduction

The construction of photoinduced gratings in glass optical fibers was first introduced by Hill *et al.* [1] in 1978. A Bragg grating structure is produced in the core of a germanosilicate-made optical fiber by inducing a periodic index change by an Ar-ion laser [2]. Optical-fiber sensors are being broadly advanced as they have numerous advantages over conventional sensors such as the ability to function in hostile environments, high sensitivity, resistance to electromagnetic interference, and perspectives for multiplexing. In recent times, there has been growing attention paid to the embedding of optical fiber sensors in composite materials for the measurement of strain, temperature, and vibration in structures such as spacecraft and airplane wings [3]. Interferometer-based fiber optic sensors have been implemented in a wide range of applications since 1980 [4]. The Fabry-Perot interferometer (FPI) was invented by the physicists Charles Fabry and Alfred Perot who published their most significant article in 1897 [5]. Fabry-Perot (FP) interferometric sensors are very promising among numerous optical fiber sensors proposed in recent times, as they are precise, simple, versatile, responsive, and immune to environmental noise. Optical sensor-based FPIs have been extensively studied due to their tunability and potentiality for signal “amplification” (*i.e.*, resonance). However, the difficulties in device fabrication have limited their commercial growth [6]. The Fabry-Perot-type sensors are usually fabricated using air-glass reflectors, in-fiber Bragg gratings, or through semi-reflective splices. Two broad types of FPI fiber sensors are identified in the literature: intrinsic, and extrinsic. This review aims to introduce some significant developments in all types of FPI fiber sensors. FPI optical fiber sensors have been used in several applications in different fields such as aircraft jet engine monitoring where inflammable materials and high voltage electricity exists, smart structure monitoring, seismic and sonar applications, the oil industry, downhole measurement in oil wells, fiber optic gyroscopes for navigation purposes, acquiring information from small complex structures, biomechanics and rehabilitation engineering, and biological and chemical sensing.

Fiber optic interferometer sensors have been developed by numerous researchers in many ways considering the improvement in functionality, efficiency and the potential implementation in sensing applications such as, Lee and Taylor who demonstrated a FPI fiber-optic sensor using a light emitting diode (LED) as optical source. The LED is used because of its low coherence emission [7]. A fiber-optic Fabry-Perot (FP) temperature sensor developed by Tseng and Chen can distinguish between temperature increases and falls and the direction of temperature difference can be determined as a time function [8]. Some FPI fiber optic sensors have been fabricated/improved by embedding in epoxy and also submerging in water for evaluating the ultrasonic sensing performances [9], by micromachining technology with a Si_3_N_4_/Si0_2_/Si_3_N_4_(N/O/N) diaphragm for pressure sensing [10], with a vortex-shedding flowmeter for the measurement of liquid flow velocities in a pipe [11], by creating a low finesse Fabry-Perot cavity between the end of a polished fiber tip for displacement sensing [12], by low-finesse FPI to generate fringes with good visibility [13], with a patch-type extrinsic Fabry-Perot interferometer (EFPI) in order to conquer interferometric non-linearity, applied to the active suppression of flutter to reduce the amplitude of the flutter mode as well as increase the speed [14], based on a nanointerferometric optical cavity by the novel ionic self-assembly monolayer (ISAM) technique for humidity sensing [15], by a three-wavelength passive quadrature digital phase-demodulation scheme with low-coherence [16], by fusing several fibers with different core diameters for use in harsh gamma-radiation environments [17], by depositing a partly reflective dielectric or metallic coating on the tip of a fiber-optic, or a glass or polymer planar substrate, and a close to entirely reflective coating on a polymer film spacer to create the mirrors of the FPI sensor [18], by bonding the silica fiber, with the ferrule, the tube, and the diaphragm together to form an interferometer with a sealed cavity for detecting acoustic emissions [19], using a technique silicon-to-silicon anodic bonding or a polymer structure with SU-8 on silicon wafer [20] a MEMS structure for pressure sensing [21], by depositing polyaniline and Nafion layers on the face of sensor head for ammonia gas sensing [22], by cascading a single-mode fiber (SMF), a photonic crystal fiber (PCF), and a hollow optical fiber (HOF) for high-temperature sensing [23], by a suspended core between two single mode fibers for a Fabry-Perot refractive index (RI) sensor for low temperature sensitivity [24], by using a diaphragm based on a polymer material for acoustic sensing [25], by the measured sample and the exteriors of a sensing fiber end for optical glass RI measurement [26], by a miniature all-silica fiber optic EFPI sensor with an embedded Fiber Bragg grating reference sensor element to determine temperature and pressure [27], by a thin film polyvinyl alcohol (PVA)-coated SMF tip for extreme temperature sensing [28], by an SMF with a Metglas (Fe_77.5_B_15_Si_7.5_) wire-based magnetostrictive transducer [29,30] or, a magnetic fluid [31,32] to measure magnetic fields, and by coating with a thin film of SU-8 photoresist and dipping into a nano-magnetic fluid for measuring magnetic fields [33].

In addition, a number of FPI fiber-optic sensors and their performance have also been reviewed in this article such as a FP cavity constructed by aligning two fiber endfaces in a hollow-core fiber in an EFPI fabrication [34]. A multiple path-match technique is used for absolute phase measurement in an EFPI sensor [35]. The necessity of tiny sensor heads for point measurement is of supreme significance in many applications. A FPI with a low-finesse and tiny cavity is a smart option for the fundamental sensing component. From an application point of view, it is very useful because of its compactness and straightforwardness [14]. Several fiber-optic sensors derived from the Micro Electro-Mechanical Systems (MEMS) technology have been proposed earlier [36,37]. The use of MEMS technology is more preferable due to its possible vastly economical manufacturing and inexpensive products. A composite cavity-based fiber optic Fabry-Perot (CCFOFP) strain sensor can be fabricated using an electrical scanning mirror and a fiber optic Michelson interferometer in which the improvement of the dynamic measurement range and multiplexing capability are observed concurrently when evaluating with the EFOFP strain sensor [38]. A two-mode interferometric sensor is designed using photonic crystal fiber (PCF) for the measurement of ultra-high temperatures (≤1,000 °C) [39]. A hybrid fiber-optic sensor is constructed by combining FPI, and MI sensors [40] with an asymmetric dual core micro-structured fiber [41]. A dual-core microstructured fiber is used to form two parallel FP cavities with low finesse between its endface and region of splicing [37]. A PDMS-based polymer-based in-plane silicon Fabry-Perot interferometer was constructed for chemical sensing [42]. A non-contact vibration sensor is fabricated from an SMF extrinsic Fabry-Perot interferometer (EFPI) and the extracted wavelet transform optical data is employed for developing a novel signal decoding technique to overcome the limitations of demodulated signals caused by complex fringe and phase ambiguity [43]. It is found in [39] that the two-mode interferometer sensor head requires an extended burn in terms of thermal annealing to attain its steady state and a sufficient level of functionality regardless of being robust and compact.

To the extent of the authors' knowledge, there are a few review articles that have partly talked about the common fabrication, sensing technologies and measurands of Fabry-Perot Interferometric fiber-optic sensors, including [44], which only covers microcavities that play a significant role in forming FPI, vibration sensing in [45], strain measurement in [46], acousto-ultrasonic sensing in [47], and also includes a number of recent reviews given in [48] where the recent trends of FPI fabrication, methods and application are presented. Reference [49] explains RI sensing, and in-line fiber optic FPI formed by using SMF is covered in [50], nevertheless, they did not completely cover all the latest and promising as well as previously-reported applications of FPI sensors, their remarkable fabrication processes, and operating principles, which provides a reason to conduct a comprehensive study of FPI sensors. Although there are some books [51,52], on fiber optic sensors which detailed a general study of fiber Bragg gratings and fiber optic sensors, including some applications of FPI sensors, this review article intends to offer a complete summary of the fabrication methods, sensing applications, and operation principles of FPI sensors in terms of the advances carried out up to recent days as well as the current status of progress in related research from all over the world. We believe that this study will definitely enhance readers' understanding on the state of the art of FPI optical sensors and their applications, also will offer better ideas for conducting further research in this exciting area. The following sections are presented in a chronological manner.

## 2. Sensor Fabrication Methods Using Fabry-Perot Interferometers

Several varieties of optical fiber have been used for the development of the FPI sensors. Yoshino *et al.* fabricated a FPI sensor using SMFs by optically polishing and coating the two end faces with a multilayer of dielectric films based on a method called vacuum evaporation [53]. A single two core fiber has been employed to develop Fabry-Perot interferometric sensors for concurrent comprehensive measurement of temperature and strain. This FPI is made up by of a pair of low reflection Bragg gratings that are holographically written with a time-division multiplexing (TDM) technique [53]. Various interesting and challenging FPI fabrication methods can be found in the literature. Authors have managed to categorize some of the significant methods into two groups: fabrication of FBI sensors with and without splicing methods. Some selected methods are presented below in the following section.

### 2.1. Optic Fiber FPI Sensors without Splicing Method

#### 2.1.1. Coherence Multiplexing Technique for Remote Sensing Based on FPI

**Figure 1.**
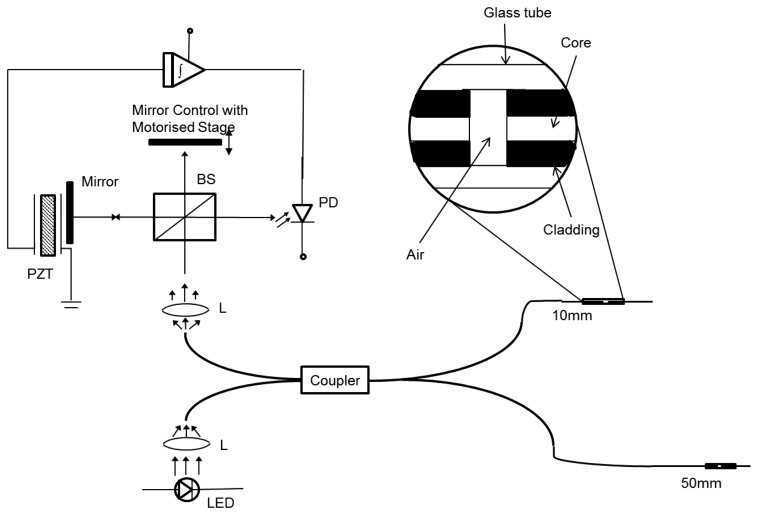
Experimental arrangement of the coherence multiplexing technique for remote sensing based on Fabry-Perot interferometers [4]. The abbreviations used in figure are light emitting diode (LED), fiber directional coupler (DC), fiber Fabry-Perot (FFP), beam splitter (BSD), photodiode (PD), integrator (mittpiezoelectric transducer (PZT).

The unbalanced FPI using frequency division multiplexing (FDM) technique is illuminated by modulating the frequency and appropriate amplitude is selected to force all the interferometer over an integral and dissimilar number of fringes. Then, for every sensing component a pseudoheterodyne carrier is developed that involves crosstalk. Furthermore, utilizing FPIs, as proposed by Dakin *et al.* [54], leads to the same problem. A coherent signal is generated in the image plane due to highly scattering objects and shows similarity with that of sample depth within the length of coherence is referred to as “crosstalk”, which is critical for the lengths for several sensors and limits the number of sensors used. Consequently, a Fabry-Perot fiber-optic sensor with coherence multiplexing is constructed to overcome this drawback [4]. A super-luminescent diode source is used to inject the light into two Fabry-Perot sensors. These FP sensors are created inside a compact fitting capillary tube by cleaving a single-mode optical fiber. Each path length of the Fabry-Perot sensors as well as the difference between the paths length are larger than the length of the source's coherence. A bulk optic Michelson interferometer is used to detect the interferometric fringes by sequentially matching the difference of path length for each Fabry-Perot fiber. The light source coherence length can be estimated by considering the visibilities of the fringes and the translation stage at a stable velocity over the zero path length mismatches as shown in Figure 1 [4]. Another low-coherence technique for multiplexed measurements on a fiber sensor array was proposed by Sorin and Baney [55].

#### 2.1.2. Micromachining Technique

**Figure 2.**
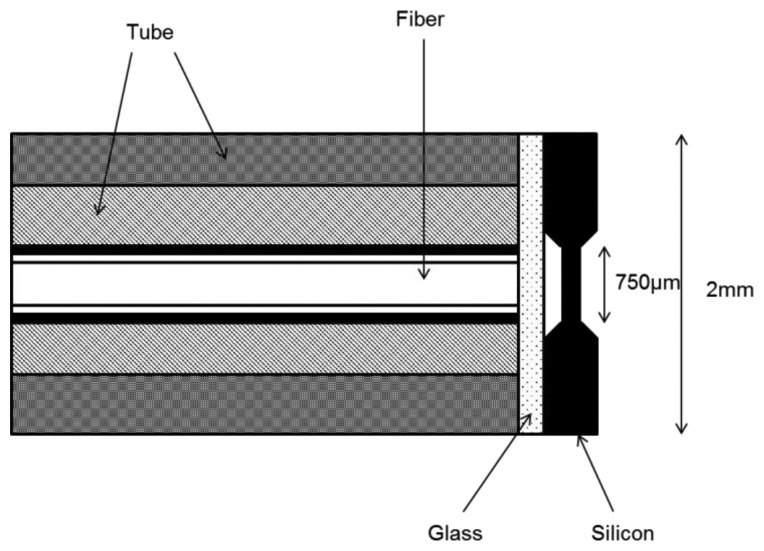
Schematic of a sensor structure. The light is sent and received through the 100 μm core fiber. The cavity length is about 7 μm and the thickness of the membrane is 8 μm [56].

A Fabry-Perot cavity is formed by two light-reflecting surfaces. The amount of light passing through the cavity depends on the partition between the two reflecting surfaces. Accordingly, if one of the surfaces is made of a membrane that deflects with pressure, the light output changes according to the magnitude of the membrane deflection. Such a strategy has been found in an FPI sensor that fabricated on a 200 μm thick-silicon wafer using the micromachining techniques (see Figure 2).

The sensor is attached to the end of a Corning Pyrex tube with 1 mm inner diameter (ID). A 6.8 μm deep etching on one side is used as a separation between the two surfaces. This separation gap is used to make it convenient to use a LED light source that is more economical, but on the other hand, the limitations of utilizing LEDs should be considered as well, such as the fact they have a much larger spectral bandwidth and their length of coherence is much shorter than that of a laser [56]. A miniature based mechanical membrane structure is fabricated on silicon wafers by anisotropic etching in potassium hydroxide. Firstly, a photolithographically-structured window is etched to a 178 μm depth on one side after the specific alignment of the masking patterns equally on each side of the silicon wafer. Then, both sides are etched to a 7 μm depth, which yields the required cavity depth and the membrane thickness of 8 μm.

The upper side of the membrane in a (100) crystal plane is polished to optical quality where the reflection should occur. Finally, a vent hole is created in the micromachined cavity to avoid any obstruction at a higher pressure caused by any resistance from the compressed air that is otherwise trapped in the cavity [56].

A micromachined Fabry-Perot interference based microcavity structure for pressure sensing was studied in [57] as well. Based on the same technique, Boyd *et al.* developed a fiber optic sensor based on FPI and it has been patented [58].

#### 2.1.3. EFPI Ultrasound Sensor Using a Thin Polymer Film

**Figure 3.**
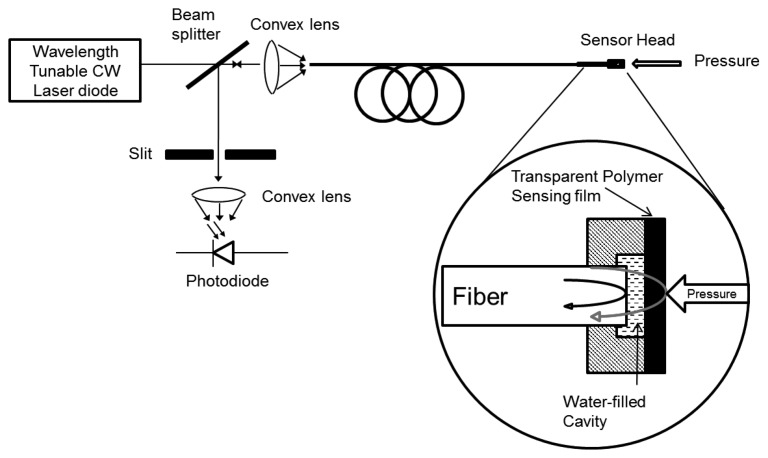
Schematic diagram of an EFPI ultrasound sensor [59].

An FPI is fabricated using a slim transparent polymer film that acts as a low-finesse FPI for ultrasound sensing. For the reason that the polymer film itself is an interferometer, shorter path length, low sensitivity to pressure and thermal differences result from the use of a slim polymer film as a FPI sensing component. As a result, phase-bias-control and complicated polarization systems are not essential. A wavelength-tunable laser diode is used as a laser light source and the laser light is launched into a multimode optical-fiber down-lead (see Figure 3). Fresnel reflection coefficient is significant parameter for interferometeric mirrors, is related with mismatching between the refractive indices of the surrounding media and both sides of the sensing film, so there is no need to apply a reflective coating on the sensing film. When the acoustic waves are applied to the sensor, the optical phase mismatch between two reflections is induced due to modulation of the film and consequently an optical intensity modulation is induced, which is then reflected back through the fiber for detection by a photodiode (PD). The sensor is controlled and maintained at quadrature by tuning the laser diode wavelength for the best possible linearity and sensitivity. The cavity that is the gap between the fiber end and the sensing film is filled with water for a couple of reasons: first, it gives the best possible fringe visibility of unity, the reflection coefficients on each side of the film will be the same and second, it gives more acoustic-impedance matching between the fiber end and the sensing film, whereas on the other hand, it will distort the uniformity in the frequency response of the sensor [59]. Similarly, the low-finesse FPI technique has used for strain sensing with some modifications [60], and has been patented [61–63].

#### 2.1.4. Ionic Self-Assembly Monolayer (ISAM) Technique

The ionic self-assembly method (ISAM) was first proposed by Arregui *et al.* [15] for the fabrication of nano-FPI to be used as a humidity sensor. This is a well-recognized technique and has been implement for a long time in the fabrication of various thin film materials on substrates of numerous shapes and sizes. This method is based on the principle of the electrostatic force of attraction between the opposite charges of molecules of thin film deposited on the substrate. In [15], an optical fiber, treated as substrate, is cleaned and processed to induce surface charges. Afterwards, the processed substrate is dipped into the solutions of oppositely charged polymers, alternately, to produce a multilayer thin film.

**Figure 4.**
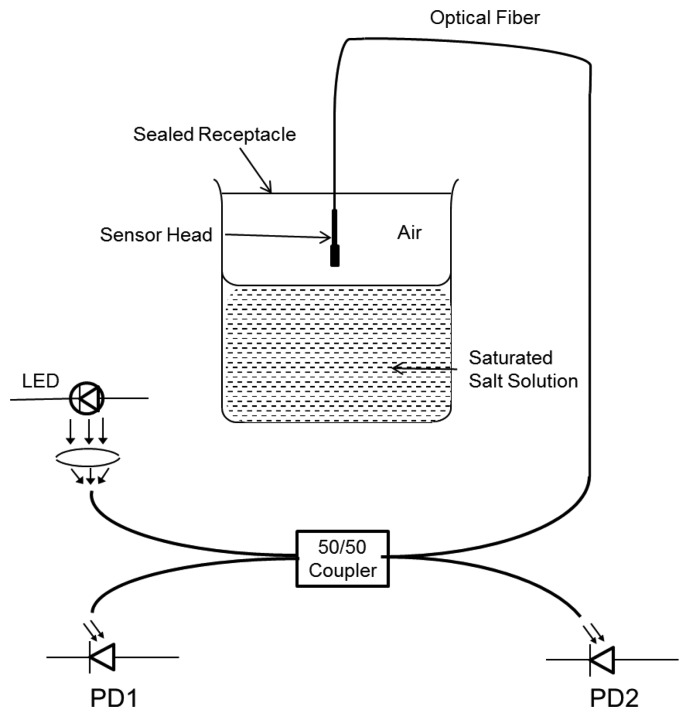
Experimental humidity sensor system design [15].

Arregui *et al.* used a solution of sodium 4-styrenesulfonate for making the anionic electrolyte in order to fabricate a humidity sensor [15] whereas poly R-478 (anthrapyridone chromophore) is used in [64] to make the sensor response fast based on time. The Au:PDDA cationic electrolyte is formed by a solution of gold colloids protected with a polymer coating of poly(diallyldimethyl ammonium chloride) (PDDA). Using the aforementioned solutions, a bi-layered structure *i.e.*, [Au:PDDA+/PSS‐]_n_, is fabricated at the fiber end by the ISAM process, where n denotes the total number of fabricated bilayers. The humidity sensor setup is shown in Figure 4, arranged after the build-up of the coating of [Au:PDDA+/PSS-]_n_. Two photodetectors are used to monitor the reference optical power for the incident signal at the coating, and other measures the reflected optical power. For observing the interferometric phenomenon, a LED source is used instead of a laser source for the same reasons stated in [56]. The humidity sensor is set up via a small hole through a sealed receptacle as shown in Figure 4, and hung in the air near above the given solutions for humidity sensing [15].

#### 2.1.5. Langmuir-Blodgett (LB) Technique

An alternative to the ISAM process *i.e.*, the Langmuir-Blodgett (LB) process is also presented in [65] for the synthesis of multilayer films having thicknesses on the order of nanometers. A submicrometer FPI is fabricated by using the LB technique which allows the thickness management at the molecular level and the layer-by-layer deposition of multilayer structures. The FPI fiber-optic cavity can also be fabricated from a single chemical species by this technique. Such homogeneity of fabrication is unattainable with other methods, for example, the ISAM method which requires alternate layers of materials, charged oppositely.

**Figure 5.**
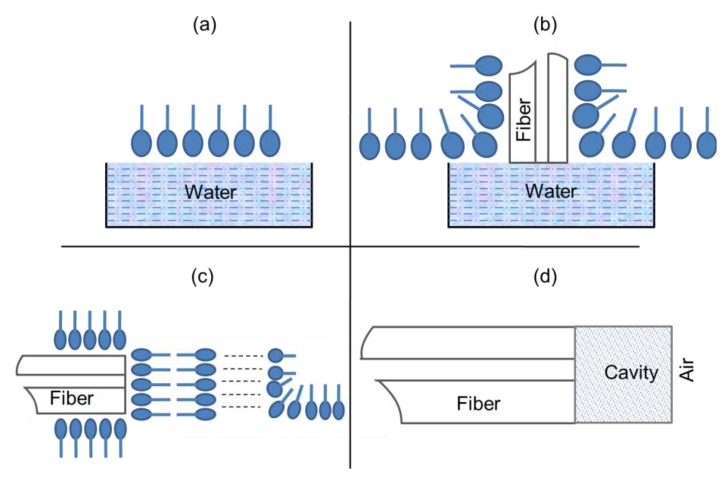
Illustration of the Langmuir-Blodgett method. (**a**) Formation of a monolayer film of aliphatic molecules on the surface of water, represented by hydrophilic circles and hydrophobic rods; (**b**) deposition of one layer on the optical fiber by passing up through the film; (**c**) after depositing the six layers on the fiber end through the film, deposition of a 7th layer; (**d**) formation of the cavity at the fiber end of the fiber with patterned refractive indices [65].

Tricosanoic acid [CH_3_(CH_2_)_21_CO_2_H] is used as the cavity material for this technique. The acid is spread using a dilute chloroform solution with 0.1 mg·cm^−3^ density, over the pure water surface that is the subphase of one compartment of a Nima Technology LB trough, and left for approx. 10 min at a temperature of ∼20 °C and after that it is compressed at the rate of 0.5 cm^2^·s^−1^ and deposited vertically with a rate of 1 cm/min onto the fiber end at a certain surface pressure, *i.e.*, 30 mN·m^−1^. The cleaved face fiber is perpendicular to the surface plane of the film. Y-type structures are fabricated passing the fiber end through the film where the amphiphilic molecules are rearranged in head to head structures and tail to tail structures as shown in Figure 5a–c. The deposition of multilayers using the LB technique on the fiber end forms a cavity where the first mirror is formed at the optical fiber and LB interface and the other mirror is formed at the LB film and air interface, as shown in Figure 5d.

#### 2.1.6. Focused Ion Beam (FIB) Milling Technique

FIB milling is applied on the end of a fiber to fabricate an all-glass FP modal interferometer (FPMI) as an alternative technique for fabricating ultra-small, micro-cavity sensors with an air-gap based on FPIs for relatively higher temperature sensing [66]. FIB milling is an ideal technology for nano-fabrication and micro-machining because of its tiny and convenient spot size (on the order of a few tens of nm). A micro-cavity can be fabricated in order of sub-wavelength fiber, while the higher current density of an electron beam is used during FIB milling. A micro-machined fiber end with tapered structure can be developed by an SMF with splicing with the MMF method which can be used as a highly sensitive modal interferometer. A micro-notch cavity is machined directly on an SMF tapered end in order to fabricate a tapered FPMI sensor. The cavity measures a few micrometers in height, width, and length. The sensing can be measured by the reflection of light from the end-faces of the cavity. It is reported that the temperature sensitivity of proposed sensor is about 20 pm/°C.

The fabrication of an optimal fiber tapered end structure can be done by using a commercially available pipette puller with optimized pulling velocity and CO_2_ laser power. The tapered end is coated with an aluminum (Al) layer of 150 nm thickness through vacuum evaporation deposition. Accumulation of gallium ion is prevented by this Al layer in the FIB micromachining process. Subsequently, the Al-coated fiber tip is located steadily in the FIB milling chamber with a conductive tape. In the end, the Al layer is totally removed by dipping the micro-machined tapered fiber end in an acid such as hydrochloric acid (HCl), for 30 min, then deionized water is used to clean it properly to make it ready for use.

### 2.2. Optic Fiber FPI Sensors with Splicing Method

#### 2.2.1. FPI with Dielectric Mirrors by Standard Fusion Splicing Techniques

Dielectric mirrors are used in continuous lengths of single mode fiber to formulate reflectively monitored Fabry-Perot interferometers by the fusion splicing technique for use as wavelength and temperature sensors [57]. Electric arc fusion is applied for splicing two single mode fibers, one of whose ends is coated with a ∼1400 Å TiO_2_ layer. The splicing is controlled at lower values of arc current and a shorter period of time than usual, thus several splicing pulses are required to construct each mirror. A pulsed light source is used for monitoring the reflectance throughout this process. The reflectance is found to diminish with every splicing pulse, followed by an initial increase and the fabrication of that mirror is completed when the required reflectance is attained. The implementation of this sensor for measuring temperature and strain can be found in [3,67].

#### 2.2.2. Semi-Reflective Fusion Splice Technique

A 10 mm in length strain sensor is fabricated and placed on a cantilever beam surface for the formation of all-fiber FPI strain sensor using a simple semi-reflective fusion splice technique [68]. Two cleaved fiber ends with York 3.5 μm/125 μm specifications are coated with silver by dipping into a Rochelle solution for one hour. Then both of fiber ends are spliced with a fusion splicer by applying a sequence of fusion cycles until the desired reflectivity has been achieved.

**Figure 6.**
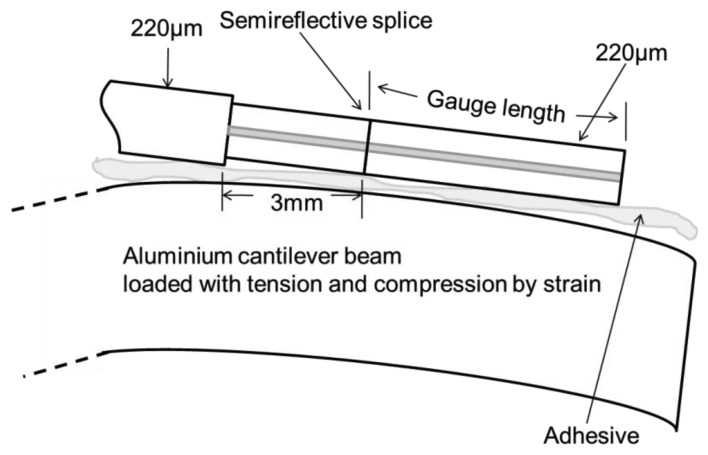
Detail of a Fabry-Perot strain gauge placed on the surface of a cantilever beam [68].

The FPI cavity is fabricated by cleaving and silvering the fiber end that is 10 mm away from the splice. The other FPI mirror is made highly reflective, instead of being matched with the first. A cleaved and silvered fiber end gives a distinctive reflectivity of 0.8 that brings up the notion of semi-reflection. The Fabry-Perot strain sensor is attached to an aluminium beam surface by using cyanoacrylate adhesive as shown in Figure 6. The beam is considered as a cantilever and eventually, it is loaded with tension and compression through a strain having a range of 1000 με; the phase-strain relation has been found to be linear and as well as symmetric with respect to tension and compression [68].

#### 2.2.3. A Miniature Fiber-Optic Fabry-Perot Interferometric Modulation Technique

Two pieces of HiBi fiber are reflectively spliced with each other and then one of the given HiBi fibers is cleaved closer to the point of splicing for fabricating a miniature FPI temperature sensor with a length of ∼3 mm. The half waveplate and the first beam splitter are positioned by a selective fiber coupler to make the system more practical and easy to construct. The interferometric phase change of the carrier is measured whereby the fiber sensing gives a gradual shift in temperature ranging from ∼23 °C to ∼32 °C [69].

The cavity of a low finesse FPI sensor is fabricated by two parallel mirrors with less reflectivity and a fixed separation distance [70,71]. A number of signal processing techniques are proposed to support sensing measurements. However, all these techniques suffer from phase mismatch because of multiple reflections which occur at the boundaries of two reflecting surfaces. In order to overcome this problem, a passive signal processing technique has been proposed for miniature FPI sensors as in [70], which is based upon phase stepping methods and to achieve four outputs with phase shifted intensities, the adjacent axial modes are used by the multimode laser diode.

A high-temperature sensor using a miniature FPI is constructed with short hollow-core fiber (HCF) with a piece of an SMF by fusion splicing. The used lengths of HCF and SMF in the reported work are given as 70 μm and 510 μm, respectively, which are indeed tiny [23]. A micro-cavity FPI and a Mach-Zehnder interferometer are constructed by splicing two SMFs to both ends of a piece of PCF fiber to form a strain sensor [20].

#### 2.2.4. Microscopic Air Bubble FPI by Simple Splicing Technique

A monolithic FPI that consists of a microscopic air bubble is constructed by splicing the SMF-28 with the index-guiding PCF using an arc-discharge splicing technique. A non-commercial PCF is used with an air hole diameter of 2.3 μm arranged around the core in a hexagonal-like pattern with an outer diameter of PCF of 125 μm to make easy the alignment with the SMF [72].

Arc-discharge is used to heat the fiber ends above the softening point for the short interval of time during the splicing. After that the fibers are pushed collectively to form an enduring joint. The softening point of PCFs is normally lower than that of all-solid SMFs because they have many microscopic holes in the cladding. Hence, the temperature reached through the arc discharge will be highly adequate to go beyond the PCF softening point when the default splicing parameters are set. Under these conditions, the holes of PCF will diffuse completely with each other. Consequently, some amount of air remains inside the space which produces the microbubble and its position can be set at the center of the fiber if the holes inside the PCF are completely symmetric [72]. A similar micro air bubble is formed with a SMF and a silica tube by the fusion splice method to fabricate an ultrahigh sensitivity FPI pressure sensor [73].

#### 2.2.5. Two-Mode Interferometric Sensor by Fusion Spliced Technique

A conventional program for splicing with an arc-discharge machine is considered to fabricate a two-mode interferometric sensor based on a short piece of a home-produced PCF. The PCF is fusion spliced on both sides to standard optical fiber (Corning SMF-28e). During splicing, part of the holey structure of the PCF collapsed (for a length of about 300 μm) due to the heat required to fuse the two fibers. The guiding structure of the PCF no longer exists in this collapsed region, and it is replaced with an all-silica rod. The first collapsed region is utilized to excite two modes in the PCF. The fundamental SMF mode has diffracted in the pure-silica region, and as a result the mode broadens. This allows the excitation of two modes inside the PCF due to the mismatch between the broadened Gaussian mode and that in the PCF. An interferometric pattern is created while such modes propagatd with diverse effectual indices, and accordingly, with diverse phase velocities, through the uncollapsed PCF region, until they recombine, through the second PCF collapsed zone, with the fundamental Gaussian mode of the second piece of SMF [39].

#### 2.2.6. MEFPI Sensor by Chemical Etching Technique

A micro-extrinsic Fabry-Perot interferometer (MEFPI) sensor with a cavity length given as ∼9 μm can be fabricated by chemical etching of commercially available erbium (Er)- and boron (B)-doped fibers and then splicing with an SMF. The etching procedure is performed for about 40 to 45 min using a solution of a mixture of 20 mg NH_4_F, 30 mL of buffered hydrogen fluoride (BHF), 40 wt% of HF and 54 mL of deionized water. Then a simple splicing method is applied between the etched EDF and BDF to an SMF (Corning, SMF-28) to fabricate a micro sensor based on FPI. This type of sensor is fabricated for the measurement of strain or temperature, but not both simultaneously [74].

#### 2.2.7. Chitosan-based Fabry-Perot Interferometry

**Figure 7.**
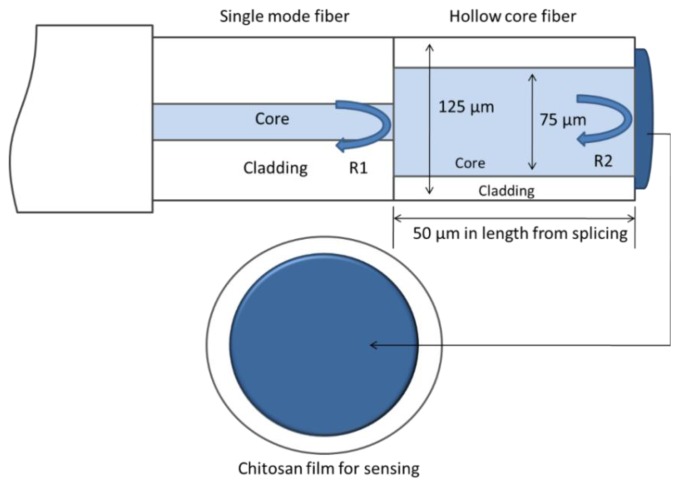
The model diagram for the chitosan-coated FPI, RH sensor [75].

This miniature FPI humidity sensor is constructed by using an SMF, in which a hollow core fiber with a physical length of 50 μm is used and a thin layer of natural polymer *i.e.*, chitosan, sensitive to moisture (humidity) is coated over the end of the hollow fiber as shown in Figure 7.

For this fabrication process, 1% conc. of chitosan is chosen to synthesize the chitosan film because such a quantity of chitosan is found to produce the most perfect diaphragm by forming a thin and flat coating on the fiber end which gives an advanced permeability because of the diminution in crystallinity. Before splicing, the SMF-28 is first cleaved by a cleaver with high accuracy in order to achieve the best quality of the fiber end that provides reflectivity of ∼4%, for fabrication of the sensor. In order to form a micro gap, a hollow core fiber (Polymicro TSP050150, outer diameter-OD: 125 μm, inner diameter-ID: 75 μm) is then spliced with the well-cleaved SMF end using a conventional fiber splicing device with an arc power of 8 bits, gap 23 μm and arc duration of 300 ms. Then a cleavage of the hollow core fiber is performed under the microscope in order to achieve the desired cavity length. Finally, the SMF with microcavity and 1% chitosan solution are located inside a drying chamber with a humidity of 9% RH and a temperature of 25 °C to perform the dip coating procedure, which gives a sensing diaphragm of thin chitosan over the HCF [75]. Suspended chitosan substrate film was used in a recent study on FPI fabrication to form an immunosensor [76].

#### 2.2.8. Femtosecond Laser Micromachining and Fusion Splicing

**Figure 8.**
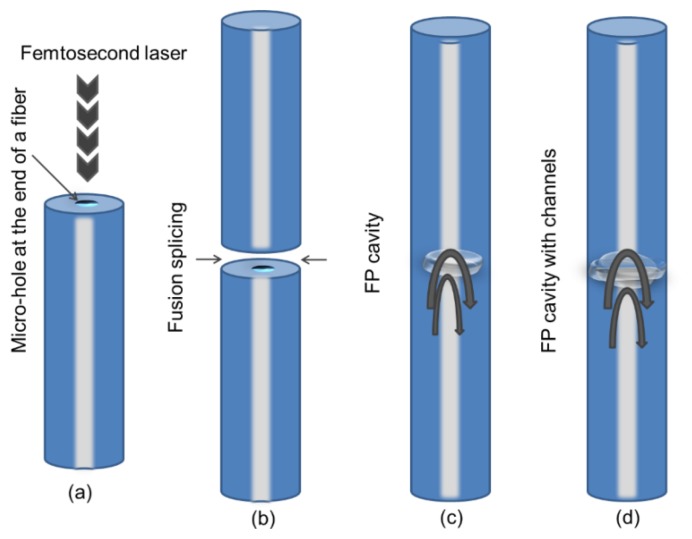
Illustration of fabrication of a FPI cavity inside the fiber [77]. (**a**) The creation of microholes, on the order of ∼1 μm using a femtosecond laser, through the center of the fiber core. (**b**) Splicing of the two fiber ends with microholes. (**c**) Formation of the FP cavity. (**d**) Introducing the vertical cross-through microcavity for the fabrication of microchannels.

An easy fabrication of an FPI cavity sensor for RI measurement is presented in [77]. Microholes, on the order of ∼1 μm in diameter, are created using a femtosecond (fs) laser, through the center of the fiber core and then splicing of the two fiber ends with micro-holes is carried out to make a FP cavity where the fusion current is fixed as 16.3 mA for a duration of 2.0 s. Then a hollow sphere with a diameter of ∼60 μm is formed. In the end, FP cavity is micromachined by a fs laser to make a micro-channel with a gap of ∼38 μm, which can be used for RI sensing of the given liquid interacting inside or outside of the cavity as shown in Figure 8 [77]. In another work, a fs laser is used to fabricate an FPI sensor by producing a diaphragm-like thin fiber piece at the end of a fiber [45].

A micromachining technology is also used in [10] to develop a FPI pressure sensor with high sensitivity by using a Si_3_N_4_/Si0_2_/Si_3_N_4_(N/O/N) diaphragm with anisotropic etchant solution *i.e.*, KOH(aq). A pair of SMFs with ends coated by TiO_2_ film are used to fabricate a FPI based on the fusion splicing technique. Typically, 10 to 20 splicing pulses with low arc current are used until the desired value of the mirror reflectance is achieved. The splicing to the next fiber optic mirror is carried out with the first fiber after cleaving with the desired cavity length, to make the FPI sensor [10].

## 3. Operating Principles

It is clear that the FPI sensor consists of a cavity between two semi-reflective surfaces or one that is semi-reflective and the other is a full-reflective surface, so the total reflection will be the result of two reflective powers, *i.e.*, R_1_ and R_2_, it can be expressed by [78]:

(1) $$P_{r}=P_{i}(R_{1}+R_{2}-2\sqrt{R_{1}R_{2}}\cos\varphi )$$

where *P_i_* and *P_r_* are the incident and reflected optical powers, respectively, *R*_1_ and *R*_2_ are the reflections from reflective surfaces (*R*_1_ and *R*_2_) ≪ 1, *φ* is the phase shift of complete cycle from one reflective surface to another reflective surface and it can be written as:

(2) $$\varphi =\frac{4\pi nL}{\lambda }=\frac{4\pi \text{nfL}}{c}$$

where *n* is the RI,*L* is the total cavity length of FPI, *λ* is the optical wavelength, *f* is the optical frequency and *c* is velocity of light.

From Equation (1) it is obvious that the reflected power *P_r_* can be varied by varying the phase shift which depends on the different physical parameters described as:

(3) $$\varphi =\varphi_{\text{initial}}+\Delta \varphi_{L}+\Delta \varphi_{f}+\Delta \varphi_{T}$$

with:

(4) $$\Delta \varphi_{L}=\frac{4\pi }{\lambda }(n\Delta L+L\Delta n)$$

(5) $$\Delta \varphi_{f}=\frac{4\pi L}{c}\left(n+f\frac{\Delta n}{\Delta f}\right)$$

(6) $$\Delta \varphi_{T}=\frac{4\pi }{\lambda }\left(L\frac{\Delta f}{\Delta T}+n\frac{\Delta L}{\Delta T}\right)$$

where *φ_initial_* is the initial phase shift and Δ*L*, Δ*n*, Δ*f*, and Δ*T* are the changes in length, RI, frequency and temperature, respectively.

Equation (2) is the FPI principle equation which has been being followed by many researchers for different types of applications, *i.e.*, strain, temperature, pressure, magnetic field, voltage, humidity, vibration sensing and many more. A variety of hypotheses concerning FPI fabrication and characterization are presented in the literature. For the reader's better understanding some examples of the working principle equations of different sensors are given in this study. For magnetic field sensing, the RI of the cavity can be related to the magnetic field by [32]:

(7) $$n=(n_{s}-n_{0})\left\{\coth\left(\alpha \frac{H-H_{c}}{T}\right)-\frac{T}{\alpha (H-H_{c})}\right\}+n_{0}\text{for}H>H_{c}$$

where *H_c_* is the critical value of the magnetic field,*n*_0_ is the RI of the magnetic fluid under the critical magnetic field, *n_s_* is the saturation value of the RI of magnetic fluid, and *α* is a fitting coefficient. It is regarded that for a certain kind of magnetic fluid film, *n_s_*, *n*_0_, α and *H_c_* are constants. For a given temperature T, *n* can be easily determined by *H*.

The humidity sensor based on RI change is also reported in [79]. In this configuration, a FPI with an open interferometric cavity is coated with polyacrylamide (PAM), which is a humidity sensitive material. The RI of PAM varies when it absorbs water vapor. This induces a spectral shift Δ*λ_v_* and the relationship is given by:

(8) $$\frac{\Delta \lambda_{v}}{\Delta n}=\frac{\lambda_{v}}{n_{\text{PAM}}}$$

where *λ_v_* is the center wavelength of reflection, *n_PAM_* is the RI of PAM and Δ*n* is the PAM RI change. The distance between two interference peaks is given by:

(9) $$\lambda_{2}-\lambda_{1}=\frac{\lambda_{1}\lambda_{2}}{2n_{\text{PAM}}L}$$

where *λ*_1_ and *λ*_2_ are the wavelengths of consecutive FPI reflection peaks, *L* is the cavity length. The RI of PAM will be changed when it humidity changes, which can be used as a principle for humidity sensing. A FPI is sensitive to variation in gas RI and gas RI is a function of pressure. Based on this principle, a FPI can also serve as a pressure sensor as reported in [42].

The cavity length of a FPI is sensitive to vibration and this has been investigated in detail by Jia *et al.* [80]. A microelectromechanical system (MEMS)-based ultra-sensitive FPI sensor has been developed for acoustic wave sensing [36]. It has been demonstrated for partial discharge detection inside high voltage transformers. In this sensor, the FPI cavity length is modeled as:

(10) $$\Delta L=\frac{Pa^{4}(1-\upsilon^{2})}{4.2Eh^{3}}$$

where *P* is the ambient pressure related to cavity pressure, Δ*L* is the cavity length which actually is the deflection of the membrane, *α* is the half side length and *h* is the thickness of the membrane, *E* and *υ* are the Young modulus and Poisson ratio of the membrane material, respectively.

## 4. Sensing Applications of Fabry-Perot Interferometers

### 4.1. Temperature Sensing

FPI fiber-optic sensors are very sensitive to thermal radiation. Such a property has been employed in support of the measurement of thermal variations [53,60,70,74,81,82], and direction of temperature change [8] for a long time. Until now, the widest temperature range reported is −200 to 1,050 °C cited by Lee *et al.*, that is found through the characterization of a fiber FPI temperature sensor with internal mirrors [7,83]. It is confirmed in [23,39] that it can be used for high-temperature sensing up to 1,000 °C. Such a temperature sensor can be used to detect even minute temperature differences produced by a human body [53]. In addition, a coherence multiplexed remote FPI fiber optic sensor is used as a point sensor [67] for remote point ‘temperature change’ measurements [4]. FPI fiber-optic temperature sensors can be implemented for some significant applications such as transformers, cancer treatment where the tumor is subject to microwave radiation, and for monitoring structural materials [57] where the usual sensors are not appropriate [69]. Fabry-Perot interferometers have been fabricated/656modified in many ways for temperature sensing, for instance it can be combined with a Michelson interferometer to fabricate a hybrid temperature sensor [41]. A miniature fiber FPI presented in [28] offers a constant temperature reading with a sensitivity up to 173.5 pm/°C^−1^ at above 80 °C, whereas a temperature sensitivity of about ∼20 pm/°C is reported in [66] for an all-glass FP modal interferometer (FPMI). An all-silica in-line fiber Fabry-Perot etalon with feedback-controlled cavity length based on a piezoelectric ceramic unimorph actuator has been demonstrated for simultaneously sensing of acceleration and temperature [84]. A thin core fiber [85] is used to form a high temperature FPI sensor with a sensitivity of ∼18.3 pm/°C and it is capable of sensing temperature up to 850 °C [86]. There are other proposed FPI sensors with higher temperature sensitivities, for example ∼5.2 nm/°C [87] and ∼39.1 nm/°C [88]. Some patents on FPI based temperature sensors can be found in [89,90].

### 4.2. Mechanical Vibration Sensing

FPI vibration sensors find significant applications in geological surveys, large civil structure diagnoses (<10 Hz), inertial navigation, consumer electronics, geological surveys, oil/gas field exploration, and earthquake monitoring (<20 Hz) [91]. A low finesse FPI vibration sensor cavity with a reported sensitivity of 9 mrad Hz^−1/2^ has also been investigated [53]. For the measurement of periodic and non-periodic vibrations, a dual-cavity EFPI is used [14,65]. Besides, a non-contact self-calibrated FPI vibration displacement sensor system [80], an all-fiber FPI sensor for low-frequency vibration measurements [90], and some other FPI vibration sensors [92–94] have been developed in early studies. A method and apparatus for detecting seismic vibrations involving a micro-machined FPI sensor have been patented by William *et al.* [95].

### 4.3. Acoustic Wave Sensing

The acoustic sensitivity of a FPI varies with the fiber length. A reference arm is not required for an FFPI as an acoustic sensor, which is a great benefit and that is why it is free from any spurious signals associated with the reference arm [53]. An FPI fiber-optic sensor using a light emitting diode (LED) temperature sensor is presented in [7]. Such a device is applicable for measuring acoustic-wave pressure too. A diaphragm-based optical fiber acoustic (DOFIA) sensor was developed to measure the attenuation properties of acoustic waves in water and a ∼30° detection range was observed with an acoustic wave attenuation coefficient of 0.0626/cm [19]. Another high sensitivity diaphragm-based interferometric fiber optical micro-electromechanical system sensor was fabricated that can detect acoustic waves on-line [36]. In addition, a polymer diaphragm-based EFPI fiber acoustic sensor system was proposed in [25] and it had an acoustic sensitivity of 31 mV/Pa·A for a maximum detected frequency of 80 MHz by FPI has been reported in [96]. A multilayer grapheme diaphragm is used in the formation of a FP acoustic sensor which exhibits an acoustic pressure sensitivity of 1,100 nm/kPa and a minimum detectable pressure equivalent to a noise level of ∼60 μPa/Ha^1/2^ at 10 kHz input frequency [97]. Another fiber optic acoustic emission sensor has been reported in a patent which is particularly useful for the vibration sensing under hostile conditions [98].

### 4.4. Ultrasound Sensing

Ultrasound has been widely used for the detection of a variety of material defects, health monitoring of structures and process monitoring [9]. Piezoelectric devices are the most commonly used components for ultrasound sensing, but they suffer a common problem of non-uniform frequency response due to poor acoustic-impedance match to liquids. The development of an extrinsic optical-fiber ultrasound sensor offers comparably better sensitivity of 61 mV/MPa [59]. Another Fabry Perot (FP) polymer film sensing interferometer was constructed based on an optical ultrasound sensing method that provides excellent detection sensitivities (<10 kPa) [18]. The development of a FPI fiber optic ultrasound sensor offers the potential to use it in fiber optic “smart structure” applications [9]. An in-line FPI fabricated from hollow-core PCF exhibits a wavelength pressure sensitivity twice as high as that of FBG which is ∼7.29 × 10^−3^ nm/MPa [99]. In another work, an in-line silica capillary tube all-silica fiber-optic Fabry–Perot (ILSCT-ASFP) interferometric sensor has been demonstrated for high intensity focused ultrasound fields measurement. It has an acoustic sensitivity of 65.4 mV/MPa over a measurement bandwidth of 2.5 MHz [100].

### 4.5. Voltage Sensing

Measuring the quality and quantity of the energy product for every power exchange point is the most significant feature of the power distribution cycle. FPI optical-fiber sensors can be a high-precision and inexpensive voltage measurement technique for high power distribution systems. AC electric voltages can be measured optically by deducting the counted pulse number from the applied voltages with a FFPI fiber-optic sensor [53]. A piezo-optical voltage sensor based on FPI was proposed and demonstrated for the measurement of AC voltages from 1 to 400 V rms. This sensor can be used as an optical voltage transducer [101]. A multipoint optical fiber liquid crystal (OFLC) voltage sensor was fabricated that can directly measure an electric field up to 800 kV/m at distributed points along power lines [102].

### 4.6. Magnetic Field Sensing

An FPPI sensor is used for DC magnetic field measurement that is considered a sophisticated method. It has been reported that the AC and DC magnetic fields are detected by the method of pulse counting [53]. Generally, quantization of a signal creates a measurement error in a digital magnetic field sensing scheme and such errors can be decreased by FFPI. A compact fiber-optic EFPI sensor that uses a wire-based magnetostrictive transducer [30] was investigated for DC magnetic field (100–35,000 nT) sensing [29]. A magnetic fluid is adopted to form an EFPI fiber-optic magnetic field sensor which produces a measurement sensitivity of 0.0431 nm/Gs [31] and 33 pm/°e [32]. A nano-magnetic fluid- based EFPI has also shown good results in the measurement of magnetic fields [33]. Another EFPI sensor is reported for DC magnetic field sensing and it has a dynamic range of 50–40,000 nT [30]. Some patents on FPI-based magnetic field sensors have been filed [103,104].

### 4.7. Pressure Sensing

Fabrication of good performance Fabry-Perot pressure sensors with lower measurement errors is a demanding issue. An FPI fiber-optic sensor has shown good response for static pressures of 15–1,000 psi by measuring the pressure-induced deflection of a membrane [56]. A N/O/N diaphragm-based FPI was demonstrated [10]. It has a pressure sensitivity of 0.11 radian/kPa and the pressure sensitivity is proportional to the area of the diaphragm. A gas pressure sensitivity of 1,526 nm/RIU was obtained by a FFPI sensor when applied to different gaseous environments [105]. In addition, some other FPI fiber-optic sensors were fabricated and tested in [10,27,37,45,57,60,106–108] for pressure measurements. An EFPI pressure sensor showed a sensitivity of 2.75 × 10^−8^ l/kPa [109]. An ultra-high FPI pressure sensor has been reported in [73] that has a sensitivity of >1,000 nm/kPa. A miniature FPI fabricated at the tip of an FBG reported in [110] exhibited a sensitivity of 0.0106 μm/psi over a range of 1.9–7.9 psi. A FPI based on PCF is used was a pressure sensor with a sensitivity of ∼13 pm/°C over a range of 0–40 MPa [111]. Some of the examples of patents on FPI-based pressure sensors can be found in [112–115].

### 4.8. Strain Sensing

Strain monitoring of materials for structures such as building frames, bridges, tunnels, and dams, and smart structures are essential throughout the lifetime of the structure [55]. A strain range of 1,000 με and 12,255 με are reported in [34,68], respectively, for strain measurement by FPI fiber-optic sensors. A FPI based on a dual-cavity was used as a strain sensor [116]. Embedding a FPI sensor in Hercules AS4/3501-6 graphite/epoxy composite specimens is advantageous for strain measurement [117]. Another FPI embedment fiber optic sensor was designed for micro-strain measurement of concrete [118]. A polymer film-based FPI strain sensor has proven to be more robust and reliable than gauges [60]. PCF enabled micro-FPIs [72] and FPI-Michelson hybrid interferometer [20,41] are more competent to provide reliable measurements during high-cycle, high-strain fatigue tests than resistive strain sensors [106,119]. A temperature insensitive FPI is constructed using a chemical etching method shows a high strain sensitivity of ∼3.15 pm/με [120]. A GIF50 fiber with a core diameter of 50 μm was used to form a FPI by chemical etching with HF that showed a strain sensitivity of 4.06 pm/με [121], whereas a graded index multimode fiber with a larger core diameter of 62.5 μm gives a lower strain sensitivity of 3.14 ± 0.05 pm/με [122]. The FPIs constructed in [7,35,39,53,67,70,83,123] can be employed as static and mechanical strain sensors too. Some patents related to strain sensing are [89,124–126].

### 4.9. Flow Velocity Sensing

There is a growing demand to precisely measure flow velocity of complex fluid flow phenomena. The FPI fiber-optic sensor is a potential candidate for flow velocity measurements. A vortex-shedding flowmeter with a fiber-optic Fabry-Perot interferometer (FFPI) was constructed for measuring the liquid flow velocity in a pipe. A linear optical modulated frequency dependence on flow velocity is found over the range from 0.14 to 3.0 mrs [11]. A multiple measurement points-based FPI sensor was implemented for spectroscopic flow velocity measurements. It was observed that with the assistance of a higher output power laser and higher finesse mirrors, the measurement uncertainty can be further reduced [127]. The fiber Fabry-Perot interferometric technique is compared with the Michelson interferometer in [83] based on flow velocity measurement and FPI has shown good agreement in flow velocity sensing.

### 4.10. Humidity Sensing

A nano-FPI humidity sensor can be fabricated using the ISAM method for achieving its operating range of 11.3% to 100% relative humidity with a very short response time; it is applicable for human breathing monitoring [15,64]. A slim layer of a moisture-sensitive natural polymer chitosan-assisted FPI sensor is proposed for a range of relative humidity from 20% RH to 95% RH. A sensitivity of 0.13 nm/% RH with a quick response time of 380 ms and RH uncertainty of ±1.68%RH was demonstrated [75]. A nanocomposite hygrometer polyacrylamide-coated low temperature sensitivity FPI sensor was proposed and it exhibited a relative humidity sensitivity of ∼0.1 nm/(1% RH) over a range of 38% to 78% RH and ∼5.868 nm/(1%RH) over a range of 88% to 98% RH, respectively [79]. In a recent publication [128], an extrinsic micro-cavity FPI with a high sensitivity of 0.307 nm/%RH over a range from 8.8% to 88.1% RH has been demonstrated. The FPI sensor mentioned in the patent [129] can be used as a humidity sensor.

### 4.11. Gas Sensing

Gas sensing by optical-fiber sensors has drawn a lot of interest for their extensive applications in the areas of the environment, industry, healthcare, battlefield, and national security. An FPI sensor with polyaniline and Nafion layers deposited on the face of sensor head was fabricated and used as an ammonia gas sensor [22,42]. A vapor-sensitive polymer layer and a silver layer are deposited in sequence on the fiber endface to construct an optical fiber FPI gas sensor. The gas sensitivity of this sensor reported for Norland Optical Adhesive (NOA) 81 and polyethylene glycol (PEG) 400 are 0.1 pm/ppm, and for methanol vapor it is 3.5 pm/ppm, respectively [130].

### 4.12. Liquid Level Sensing

Liquid level measurement with high precision is a vital necessity in chemical and fuel storage processing systems. An asymmetric FPI has been demonstrated for high accuracy continuous liquid level sensing. Its calibrated sensitivity is 2.4 mV/mm, over a range of 2.3 m (water) [131]. An optical fiber extrinsic Fabry-Perot (FP) cavity liquid-level sensor is formed with a SMF fiber and the elastic silicon layer that has produced an accuracy of 2 mm over a full scale of 3.5 m (water) under an ambient temperature range of 10–38 °C [132]. In addition, various FPI liquid level sensors have been proposed in the literature, such as a double-fiber FP cavity with a diaphragm serving as a measurement component [133], a dual-optical-fiber-sensor system consisting a Fabry-Perot (FP) pressure sensor and a fiber Bragg grating (FBG) level sensor [134], an extrinsic Fabry-Perot optical fiber interferometer with an all fused-silica structure and CO_2_ laser heating fusion bonding technology with a high sensitivity of 5.3 nm/kPa (36.6 nm/psi) [135].

#### 4.13. Refractive Index (RI) Sensing

RI measurement has gained increasing interest among industrialists due to its important role in substance detection for chemistry and biomedicine applications. As presented in Section 1, a Fabry-Perot RI tip sensor with a RI sensitivity of −11.27 ± 0.34/RI and a resolution of 2 × 10^−4^ was achieved by using the FFT technique [24]. A simple FPI reported in [26] can be used for RI measurement of several kinds of glasses and the accuracy of this device is found to be about 10^−3^. In another work, an in-line FPI sensor for RI sensing was formed by inscribing a micro-channel perpendicularly across the core axis to enable liquid flow and light-liquid interaction in the micro-channel. The sensor demonstrated a sensitivity of ∼994 nm/RIU and the extremely low temperature cross sensitivity of ∼4.8 × 10^−6^ makes it a reliable sensor in a varying temperature environment. In addition, it has low temperature cross sensitivity of ∼4.8 × 10^−f^ RIU/°C [77]. Beside micro-channels, microstructured fiber can be used in FPI fabrication due to its micro-sized holes that enable fluid flow into and out of the FP cavity. A RI sensitivity of 1,051 nm/RIU is reported at 1,550 nm wavelength [136]. FPI has been used for monitoring the RI of gases as well in [106] with a resolution greater than 10^−5^ while monitoring changes in RI. A temperature-insensitive FPI RI sensor is reported in [137] and it is particularly suitable for circumstances where the variation of ambient temperature is large. It offers a high average RI resolution of 5 × 10^−6^ for a range from 1.314 to 1.365. Another in-line FPI RI tip sensor was reported that has a sensitivity of ∼4.59/RI for RI sensing [138]. FPI sensors have been used for RI measurement in [139,140] as well. To dates, many FPI-based RI sensors have been successfully commercialized. For example, one of the products of FISO Technologies, Inc. Quebec, Canada, is a liquid-filled Fabry-Perot optical cavity sensor [141].

## 5. Chronology and Discussion

It is already obvious that we must study the chronology of FPI sensors in order to discuss the sequential development of fabrication techniques and their implementation for sensing applications, which have been discussed with detail in the earlier sections. Achieving an adequate sequential investigative structure of the literature is an element of the dilemma in terms of presenting a fraction of the research chronology. Therefore, a specific store needs to be set using diverse forms of research in terms of chronological order.

All the possible literatures considered for this study have been categorized in two types based on their fabrication techniques: First, FPI sensor fabrication including splicing and second, without splicing methods. The sensing applications listed here have been chosen from referred articles. In order to present a chronology study, we have set an approximate starting point at 1981 that has been considered based on a few of the oldest related articles. Thus, we have divided the whole period into four time slots starting since 1981 to the current period in order to present the fabrication development and implementation of sensing applications for FPI sensors. Research articles, reviews, proceedings, books, technical papers, patents, dissertations are selected for this review study according to measurands, fabrication techniques and principles applied. Articles not directly related to these areas are not included in the statistics of this section. The statistics presented below are obtained from all the papers included in the references.

**Figure 9.**
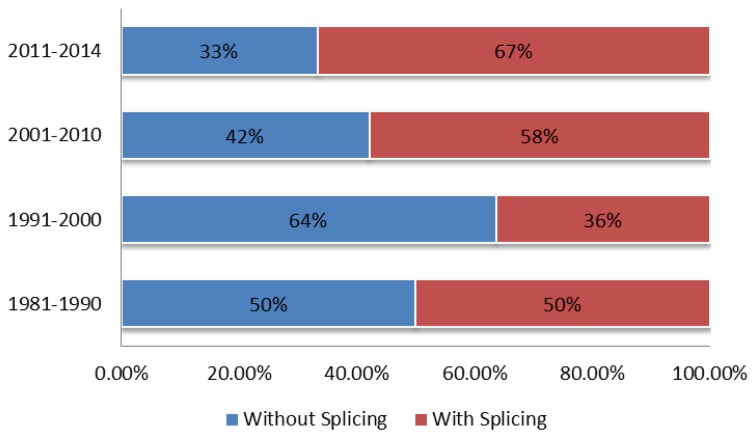
Percentages of the FPI fabrication studies in two categories are presented through some considered time ranges.

In Figure 9, an interesting trend has been investigated in terms of the percentage of the various FPI sensor fabrication techniques used over the time ranges from 1981 to 1990, 1991 to 2000, 2001 to 2010, and 2011 to 2014. The percentages are calculated over the total number of papers falling in each time range. The statistics indicate that a larger number of FPI fabrication techniques (approximately 64%) in 2011–2014 involved using splicing. Nevertheless, the use of the splicing method was quite low in 1991–2000. However, it has been gaining popularity and has become widely employed in FPI fabrication today. The reason behind this fact is that the fusion splicing technology is well developed and the splicers have become more affordable. In terms of performance, the sensors fabricated based on splicing methods are more stable and compact. Higher throughput power can be attained because of the reduced fiber cavity losses.

**Figure 10.**
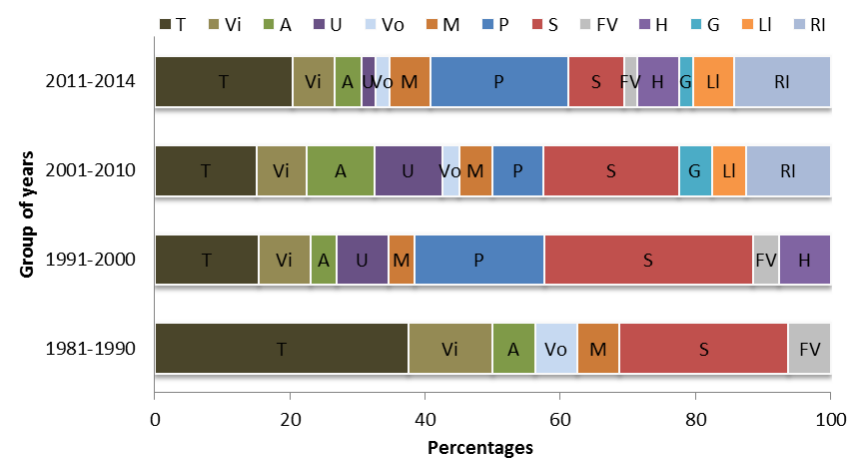
Percentage of sensing applications studied through some given time ranges. T = Temperature, Vi = Vibration, A = Acoustic, U = Ultrasound, Vo = Voltage, M = Magnetic, P = Pressure, S = Strain, FV = Flow velocity, H = Humidity, G = Gas, Ll = Liquid level, RI = Refractive index.

The percentages of sensing applications studied based on the similer strategy applied in the previous chart through the given time spans can be seen in Figure 10. Based on the sensing application, it would be hard to draw any conclusion because almost all the applications have been under consideration throughout the mentioned periods, except for a few sensing applications. It can be noticed that a lot of studies on temperature sensing using FPI sensors followed by vibration, acoustic, strain and magnetic sensing have been done over all the time ranges, whereas there is less reported research on other sensing applications because of their shortcomings such as walk-off loss, crosstalk, phase mismatch and fragility problems in the case of the tapered fiber ends used for FPIs.

Strain sensing has also been the area of interest for many researchers until 2000, then a drop of interest ocurred afterward, whereas the pressure sensing by FPI has been introduced since 1991. The most exciting statistic coming out of this chronology and review study is that, a noteworthy rise in exploration of the measurands is shown with increasing time. In recent years, there have been some growing investigations conducted for temperature, RI, vibration, magnetic, pressure, humidity, acoustic, ultrasonic, strain, velocity, and liquid level sensing using FPI sensors. These measurands have been being measured with FPI sensors for many years in civil engineering and transportation, gas and oil industries, energy industries, aerospace, biology and chemical research and other industries, *etc*.

On the basis of research being done by many researchers and also patents, fiber optic-based Fabry Perot interferometeric sensors have many appealing applications in the industrial sector. To show its significance, some of the industrial applications are given here for better understanding. As reported by Gao *et al.* [142], the real time optic fiber sensors are used widely in structural health monitoring of large civil structures such as dams, bridges, buildings and composite material structures because of their good sensitivity and other unique features. These sensors can be mounted on the surface of the structure or embedded into construction materials and structures for continuous monitoring of various physical conditions *i.e.*, damage, strain, stress, crack formation, pore pressure and temperature. The first cable-stayed bridge with a maximum span of 312 m carrying both railway and highway traffic constructed from 1997 to 2000, the Wuhu Yangtze River bridge (WYRB) situated in China is an example of such an application where some spot-weldable Fabry-Perot fiber optic strain sensors (FP-FOS) were installed on the bridge-deck sections as part of WYRB real time health monitoring system in December 2003. The sensors were more stable and worked efficiently to fulfill the monitoring requirements. Based on such efficiency, a similar monitoring sytem including FPI sensors was installed on the Zhengzhou Yellow Railway Bridge for strain monitoring as reported by Gao *et al.* [142]. Other example of fiber optic EFPI sensors are the ones embedded into the Liaohe Bridge, Liaoning, China, at the time of its construction for measuring its dynamic strain. The results obtained from the monitoring system showed the good stability and dynamic response of the sensors as reported by Zhang *et al.* [143]. Underground fault measurements have been performed using an EFPI displacement sensor in geomechanics [87]. Besides, partial discharge in transformers was detected as well by using a sulfur hexafluoride-filled FPI [144].

Kersey *et al.* affiliated with CiDRA Corporation developed and patented an FPI sensing element which can sense pressure over the range of 0–103 MPa with a resolution of 2.06 kPa and within the suitable temperature range of 25–175 °C [145,146]. The borehole pressure/temperature (BHPt) series pressure sensor developed by Sabeus Corporation can operate up to 200 °C with a measureable pressure resolution of 0.69 kPa. Some oil-gas companies, e.g., Weatherford, Sabeus, and SENSORNET offer similar products, including FPI pressure senosrs which have been used for downhole monitoring as reported by Yu *et al.* [147]. Another EFPI pressure sensor used in downhole monitoring was reported as well [109]. The other Fabry-Perot cavity pressure sensor were constructed with the pressure sensitivity of −1.8 rad bar^−1^ and typical range of 0 to 600 kPa in a turbine test application, using micromachining technique at the isentropic light piston facility (ILPF) at QinetiQ, (Pyestock, UK) [148]. It has been reported that FPI sensors offer promising prospects in nuclear power plant technology because of their unique signal processing techniques and their resistance to power loss [149]. One important application of such FPI sensors is temperature monitoring in harsh nuclear plant environments, using a sensor that has been developed by Fiso Technologies in Canada [150]. Similarly Sentec Corporation has fabricated FPI temperature sensors under a NASA contract (NAS3-27202) and ground as well as flight tests were performed successfully on an OV-1OD airplane [151].

Recently, FPI sensors have been utilized in some interesting applications in biology, the environment, and even in medical sciences. Notably, one of the applications is a biosensor that is capable of producing concentration-dependent signals from antibodies or antigens employing a FPI sensor as an immunosensor. It can eliminate fluorescence labeling or chemiluminescence which requires additional budget and labor for detecting biological interactions. Such sensors have potential for monitoring environmental pollution, in food industries, and monitoring human diseases as well [76]. Another layer-by-layer modified label-free FPI biosensor was fabricated to detect protein in real-time that also has potential for immunosensing applications [86]. One more exciting application is the use of a low-coherence FPI in constructing a surgical tool integrated with a force sensor (FS) which can be used during micro-surgery (e.g., vitreoretinal surgery) [152]. In addition, an intrinsic FPI sensor fabricated with a nanoporous zeolite thin film was used to detect dissolved organics in water [153].

**Figure 11.**
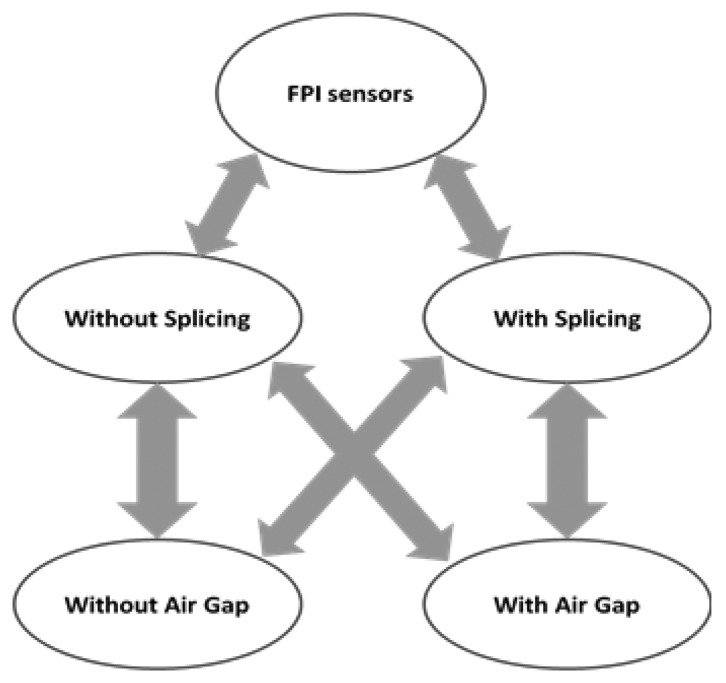
Illustration of FPI sensor categories on the basis of their fabrication.

Some of the key findings of the current review have been summarized chronologically in Table 1. This will assist target audiences (e.g., researchers, commercial industries, users, readers) to draw a quick picture of the chronological flow of the advancement of FPI fiber optics. With the categories of fabrication techniques with or without splicing, another feature has been found which is applicable for the further division such as, with or without air gap as illustrated based on entity relationships in Figure 11.

For better illustration, some of the reported techniques are given in Table 2, which summarizes all the common advantages reported in the literature based on the given time slots. This table presents the significant features of FPI sensors that make them suitable for various applications. This table will help understanding how the FPI was explored chronologically to date and proving the potential in several significant applications.

Other than the limitations presented as above in Table 2. Some usual drawbacks also have been found which require further investigation to achieve handy solutions. For example, optical spectrum analyzers (OSAs) are used widely to detect the wavelength shift, but they are relatively slow. To fully take the advantage of FPI fiber optic sensors' potential, a detector array-based spectrum analyzer can be used. Because the implementation possibilities of such sensors are endangered in some practical applications due to, for example, fragility, some useful techniques are required to encapsulate the sensor head to protect it and prevent dust particles or fluids from condensing in the cavity, as well as improving its working stability without reducing the performance. According to our knowledge, based on the literature, no work has been done so far on this aspect. Again, the stability in terms of performance, effectiveness, and extended operation has not been investigated as well as the involved components, environmental effects, and these parameters have not been considered to conquer the risk factors of FPI fiber optic sensors' implementation in practical applications.

## 6. Concluding Remarks

A review of fiber-optic FPI sensors' fabrication methods, working principles and sensing applications such as detecting temperature, pressure, ultrasonic, acoustic, as well as strain signals has been rigorously considered in order to compose an informative article keeping their pros and cons in mind. Two major categories of FPI optical fiber sensors are discussed, which are those based on fabrication method—with splicing and without splicing—whereas these types are further interrelated with reference to the air gap present inside the FPI cavity devices. Furthermore the analytical theory for all types of FPI fabricated sensors with their sensing applications to interferometry has been presented.

On the basis of the investigated study, FPI sensors have enormous practical applications so there is a need for further exploration to make these devices more efficient and beneficial as well as economical. In our perspective the special optical waveguides like photonic crystal fibers, have a chance to enable many new sensing mechanisms and configurations. Improved micro-fabrication technologies are appealing to continue the research for the enhancement of sensor performance, functionality and reliability with operating ability in harsh environments. In addition the advanced optical signal processing and network technology will enable to come up with high density fiber optic sensor networks. Unquestionably, FPI sensors will play a key role in future solutions for a widespread diversity of appliances.

It is challenging to cover all of the fabrication technologies, principles and applications in a limited length review article. Nevertheless, we have included most of the research articles, reviews, proceedings, books, technical papers, patents and dissertations that match the scope of this review study. The chronological statistics presented in the earlier section provide an approximation of the whole picture.

## Figures and Tables

**Table 1.** A brief presentation of the fabrication methods studied above with sensing applications based on the given time slots.

**Time Slots**	**[Ref.] /Year**	**Fabrication Techniques**	**with/without Splicing**	**with/without Air Gap**	**Sensing Applications**
**1981–1990**	[2] (1982)	FPI is formed by two dielectric-coated high-reflectance end faces SMF	Without splicing	With air gap	Temperature, vibration, acoustic wave, voltage, magnetic field
	[3] (1985)	A SMF is stretched through a tube so that vorex shedding can induce an oscillating strain	Without splicing	Without air gap	Flow velocity
	[4] (1988)	Coherence multiplexing remote fiber optic Fabry-Perot sensing technique	Without splicing	With air gap	Temperature
	[57] (1988)	FPI using dielectric mirrors by standard fusion splicing technique	With splicing	With air gap	Temperature and wavelength
	[68] (1990)	Semi-reflective fusion splice technique	With splicing	With air gap	Strain
**1991–2000**	[69] (1991)	A miniature fiber Fabry-Perot interferometric modulation technique	With splicing	With air gap	Temperature
	[56] (1994)	Micromachining technique	Without splicing	Without air gap	Pressure
	[55] (1995)	Low-coherence technique for multiplexed measurements	Without splicing	With air gap	Temperature and strain
	[6] (1996)	Micromachined Fabry-Perot interference-based microcavity fabrication	Without splicing	Without air gap	Pressure
	[59] (1996)	FPI ultrasound sensing with a thin polymer film	Without splicing	With air gap but water-filled cavity	Ultrasound
	[10] (1997)	FPI with Si_3_N/SiO_2_/Si_3_N_4_ diaphragm fabrication using micromachining technology	With splicing	With air gap	Pressure
	[15] (1999)	Ionic self-assembly monolayer (ISAM) technique	Without splicing	Without air gap	Humidity
	[71] (1999)	FPI cavity with low-finesse illuminated by a multimode optical fiber	With splicing	With air gap	Not studied
	[64] (2000)	Nanometer-scale Fabry-Perot interferometer by using the ISAM method	Without splicing	Without air gap	Humidity
**2001–2010**	[60] (2001)	A thin transparent elastic polymer film used as a low-finesse Fabry– Perot interferometer	Without splicing	Without air gap	Strain
	[65] (2001)	Langmuir-Blodgett (LB) technique	Without splicing	Without air gap	Not studied
	[30] (2004)	A magnetostrictive gauge and SMF are inserted in a hollow-core borosilicate tube and an airgap between these is acting as a cavity	Without splicing	With air gap	Magnetic field
	[153] (2006)	By polishing a thin layer of zeolite film on the end face of SMF	Without splicing	Without air gap	Dissolved organic matter (DOM) in water
	[123] (2007)	Two SMF is etched by acid and fusion spliced to form intrinsic FP cavity	With splicing	With air gap	Strain
	[23] (2008)	A miniature Fabry–Perot (FP) interferometricfiber-optic sensor	With splicing	With air gap	High temperature
	[72] (2009)	Microscopic air bubble FPI by simple splicing technique	With splicing	With air gap	Strain
				
	[39] (2009)	Two-mode interferometric sensor by fusion spliced technique	With splicing	With air gap	Temperature
**2001–2010**	[74] (2009)	MEFPIs sensor by chemical etching technique	With splicing	With air gap	Strain and Temperature
	[137] (2010)	FPI consisting of a segment of SMF tip coated with a SU-8 polymer thin film based on modulated Fresnel reflection	Without splicing	Without air gap	Refractive index
**2011–2014**	[111] (2011)	Spliced a short length PCF with a standard SMF	With splicing	Without air gap	Pressure and high temperature
	[66] (2012)	Focused ion beam (FIB) machining technique	Without splicing	Without air gap	High temperature
	[75] (2012)	Chitosan-based Fabry-Perot interferometry	With splicing	With air gap	Humidity
	[77] (2012)	Femtosecond laser micromachining and fusion splicing	With splicing	With air gap	Refractive index
	[45] (2013)	Thinned and roughened FPI's external surface of diaphragm by fs laser	With splicing	With air gap	High temperature
	[20] (2013)	Hybrid interferometric with micro cavity PFI and Mach-Sender	With splicing	With air gap	Strain
	[108] (2013)	Tunable micro cavity FPI by using polymer MEMS technology	Without splicing	With air gap	Pressure
	[73] (2013)	Spliced SMF with a silica tube	With splicing	With air gap	Pressure
	[31] (2014)	FPI cavity is filled with water based magnetic fluid EMG507	Without splicing	Without air gap	Magnetic field
	[82] (2014)	Miniature FPI formed by bundle-core PCF and SMF fiber by splicing	With splicing	Without air gap	High temperature
	[154] (2014)	A small segment of silica rod spliced between two SMF	With splicing	With air gap	Pressure
	[135] (2014)	Fusion bonding with a fused-silica diaphragm by CO_2_ laser	Without splicing	With air gap	Liquid level

**Table 2.** Advantages of FPI explored in the literature are presented over given time range.

**Time Slots**	**Ref. No.**	**Advantages Reported in Literature**	**Disadvantages Reported in Literature**
**1981–1990**	[53] (1982)	FPI can have high finesse; Very long distance FPI can be achieved, thus so a high spectral resolution; Walk-off loss problem has been addressed by the fabricated FPI, that involves less accuracy for the tilt angle of reflection surface than the usual form of FPI; such sensor is compatible for a scanning FPI because the optical path length in fiber can be readily modulated by appropriate external perturbations for example, temperature and mechanical forces.	The walk-off loss of light power is a severe difficulty of FPI fabrication technique that causes by the presence of tilt of the reflection surfaces. It merely decreases the effectiveness and eflectance of the conventional FPI. An increase in interferometer length is the proportion of walk-off loss increase, so construction of long-distance FPI is hardly possible.
	[4] (1988)	Crosstalk drawback is conquered. Requires no separate fiber reference arm.	Crosstalk is another serious drawback of FPI fiber-optic sensor with coherence multiplexing. A coherent signal is generated in the image plane due to highly scattering objects and shows similarity with that of sample depth within the length of coherence refers to “crosstalk”. This problem affects the lengths for several sensors and limits the number of sensors used.
**1991–2000**	[81] (1992)	FPI sensors have High spatial resolution (∼20 μm^2^), High temperature resolution (sub mK), Intrinsic calibration, High measurement bandwidth (>100 kHz), Multiplexed arrays possible, Immunity to electro-magnetic interference.	
	[56] (1994)	LED is used due to gain advantages form the proposed fabrication technique.	The limitations of utilizing LED is that, it has much larger spectral bandwidth and its length of coherence is much shorter than that of laser.
	[70] (1996)	The c technique.	Almost all the signal processing techniques suffer from phase measurement inaccuracy problem due to the multiple reflections at the two reflective surfaces of FPI.
**1991–2000**	[59] (1996)	Using a slim polymer film as one of the reflective surfaces of FPI has many advantages such as, it itself is an interferometer, shorter path length, low sensitivity to pressure and thermal differences. As a result, phase-bias-control and complicated polarization systems are not essential. Using a cavity that filled with water other than air has merits too such as, (i) giving a best possible fringe visibility of unity the coefficients of Fresnel reflection on both sides of the film will be the same, and (ii) a possibility of degrading the sensor's consistency of frequency response.	Phase-bias-control and polarization systems are essential for FPI fabrication techniques which makes the entire system more complicated.
**2001–2010**	[155] (2009)	Reasonably easy fabrication, high resolution, possibly inexpensive, and low sluggishness on temperature differences.	
	[72] (2009)	A miniature monolithic FPI with a cavity of microbubbleexhibites low-temperature sensitivity (less than 1 pm/°C) which indicates that measuring an extreme low temperature can be possible by a microcavity based FPI sensor.	
**2011–2014**	[40] (2011)	Easy fabrication even a small size of sensor heads as well as low thermal indolence, and support dielectric construction.	
	[28] (2012)	FPI can be constructed as ultra-compact in size, cost effective, and easy to fabricate.	
	[66] (2012)	It can be exceptionally tiny size, high sensitivity, all fiber connection, and particularly exclusive structure presents huge potentials for fast-response high temperature sensing mostly in miniature and harsh area with high temperature gradient. Fragility problem has not been addressed.	The fragility of tapper fiber tip is a serious shortcoming in the implementation of such sensors to sensing applications. The sensor can be broken easily due to simple handling or to vibrations that are frequently met in actual industrial applications.
	[75] (2012)	Using chitosan as reflective surfaces of FPI proposes excellent diaphragm forming capability, high-quality mechanical stiffness as well as enhanced steadiness with respect to the differences in comparative humidity.	
	[77] (2012)	A c is consistent in the measurement of exceptionally low temperature cross sensitivity.	
